# Acute hypercapnic respiratory failure in patients with obesity hypoventilation syndrome during the COVID‐19 pandemic: Four case reports

**DOI:** 10.1002/rcr2.1151

**Published:** 2023-04-20

**Authors:** Sato Nakamura, Yukio Kawagishi, Akihiro Kikushima, Atsushi Muto, Yoshifumi Suda, Kazuki Gohara, Shinichi Takeda

**Affiliations:** ^1^ Department of Respiratory Medicine Kurobe City Hospital Kurobe Japan; ^2^ Internal Medicine Kurobe City Hospital Kurobe Japan

**Keywords:** acute hypercapnic respiratory failure, COVID‐19 pandemic, non‐invasive positive pressure ventilation, obesity hypoventilation syndrome

## Abstract

Obesity hypoventilation syndrome (OHS) can cause acute hypercapnic respiratory failure (AHRF). The onset of AHRF in four patients with OHS during the coronavirus disease 2019 (COVID‐19) pandemic is reported in this study. Two men (23 and 45 years old) and two women (both 77 years old) presented to our hospital with AHRF. In the two elderly women, a prolonged supine position due to falls seemed to be the cause of AHRF. Treatment was started with bilevel positive airway pressure for all patients. While one patient died, the condition of the other three improved; they were discharged with continuous positive airway pressure. AHRF due to OHS was rarely reported in the rural region of Japan. It is suggested that increased rates of obesity due to lifestyle changes during the COVID‐19 pandemic may be responsible for an increase in the prevalence of OHS‐associated AHRF.

## Graphical abstract

The current coronavirus disease‐2019 (COVID‐19) pandemic has greatly impacted society and individual lifestyles. Recently, we encountered four patients with obesity hypoventilation syndrome (OHS) who developed acute hypercapnic respiratory failure (AHRF) during the COVID‐19 pandemic. It is suggested that increased rates of obesity due to lifestyle changes during the COVID‐19 pandemic may be responsible for an increase in the prevalence of OHS‐associated AHRF.

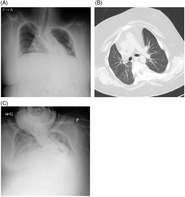

## INTRODUCTION

Obesity hypoventilation syndrome (OHS) is seen as a ventilatory dysfunction in patients with severe obesity. OHS is defined by diurnal hypercapnia (PaCO_2_ ≥ 45 mmHg) in patients with obesity (BMI ≥ 30 kg/m^2^) when other causes of hypoventilation, such as lung or neuromuscular disease, are excluded.^1^ The global prevalence of OHS is believed to have increased owing to the increase in the global incidence of obesity; epidemiological data are insufficient and the prevalence of OHS has been estimated as roughly 0.6% in the US population, based on the incidence rates of obesity, obstructive sleep apnea (OSA) in obesity, and OHS in OSA.^2^ In Japan, the prevalence of obesity with body mass index (BMI) ≥ 30 is relatively low, but the Japanese population is known to develop OSA at a lower BMI compared with other populations. The prevalence of OSA in Japan is estimated to be about 14%, and based on data showing that 2.3% of OSA patients have OHS, the proportion of OHS in the Japanese population can be estimated to be 0.3%.^2^, ^3^, ^4^ Most patients with OHS remain undiagnosed and untreated until the development of acute hypercapnic respiratory failure (AHRF).^1^ The current coronavirus disease‐2019 (COVID‐19) pandemic has greatly impacted society and individual lifestyles. Recently, we encountered four patients with OHS who developed AHRF during the COVID‐19 pandemic.

## CASE SERIES

### Case 1

**FIGURE 1:**
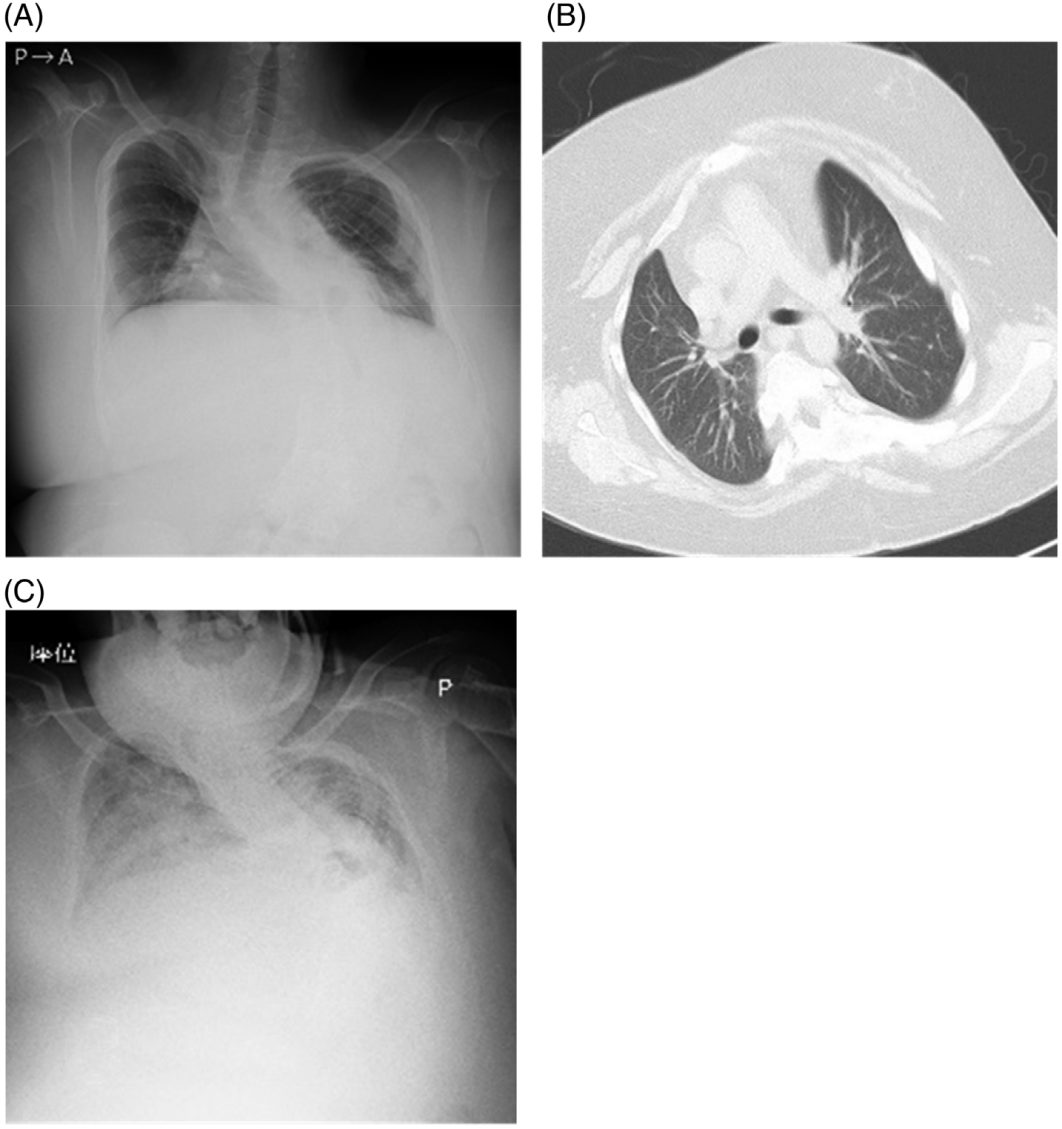
Chest X‐ray and computed tomography (CT) of case 1 (A) Chest X‐ray on admission showed the curvature of the spine with thoracic asymmetry due to scoliosis. (B) CT on admission revealed thick subcutaneous fat surrounding the thorax and no infiltration in the pulmonary fields. (C) Chest X‐ray on day 11 showed diffuse infiltration of the bilateral lungs due to pneumonia and congestive heart failure

A 23‐year‐old man with Prader‐Willi syndrome was treated for diabetes mellitus and sleep‐disordered breathing at our hospital. At the age of 15 years, he was hospitalized for respiratory failure due to obesity and began using continuous positive airway pressure (CPAP) at night. Because the working institution for disabilities he attended was temporarily closed due to the COVID‐19 pandemic, he stayed at home for 4 months during the winter and gained 12 kg in weight. In the spring of 2021, he visited our hospital with complaints of constipation, appetite loss, and dizziness for 3 days. He was 147.4 cm tall, weighed 99.0 kg, had a BMI of 45.8 kg/m^2^, and seemed lethargic and comatose. Arterial gas analysis on room air revealed AHRF (pH; 7.28, PaCO_2_; 69.0 mmHg, PaO_2_; 52.6 mmHg, ${\mathrm{HCO}}_{3}^{-}$; 30.9 mmol/L). Chest radiography showed the curvature of the spine with thoracic asymmetry due to scoliosis, and computed tomography (CT) showed no obvious opacity in the lungs (Figure 1A, B). He was admitted to the intensive care unit (ICU) and started on bilevel positive airway pressure (BiPAP) ventilation. Maximum inspiratory positive airway pressure (IPAP) and expiratory positive airway pressure (EPAP) of 20 cmH_2_O and 8 cmH_2_O, respectively, were used (mode: spontaneous/timed, back‐up rate: 12/min, inspiratory time: 1 s). On day 3 of admission, he developed pneumonia and heart failure, which improved 3 weeks after admission (Figure 1C). Respiratory status was gradually stabilized and BiPAP was completely switched to CPAP 4 weeks after admission. However, he subsequently developed critical illness myopathy and was unable to walk or stand up due to muscle weakness and numbness in the lower extremities. He underwent inpatient rehabilitation and dietary restriction and succeeded in losing 18 kg of weight by discharge. Respiratory failure improved (pH; 7.39, PaCO_2_; 48.6 mmHg, PaO_2_; 82.8 mmHg, ${\mathrm{HCO}}_{3}^{-}$; 28.9 mmol/L [room air]) and CPAP was continued at night. On day 64, he was transferred to another hospital to continue rehabilitation and subsequently admitted to a facility.

### Case 2

**FIGURE 2:**
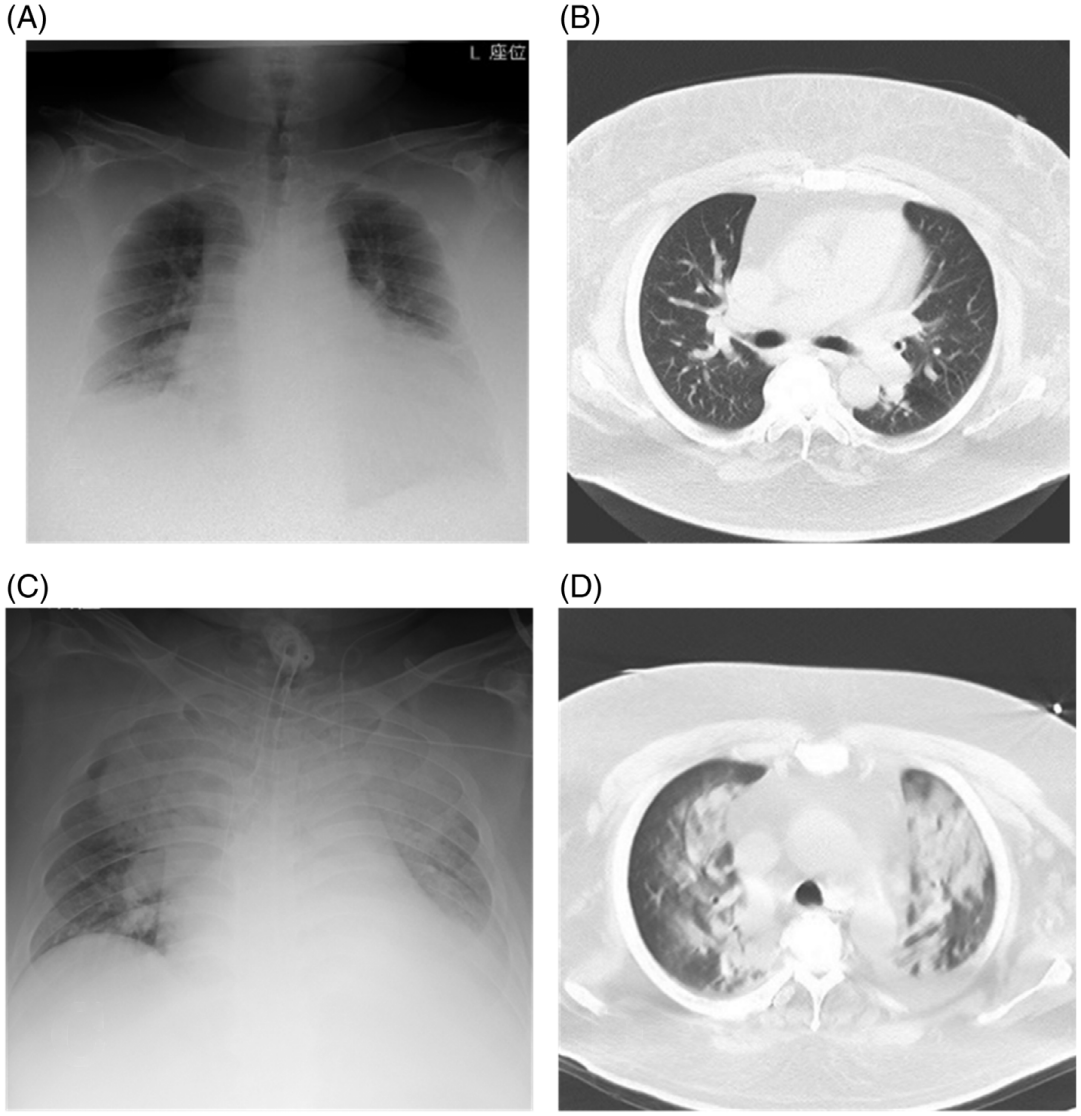
Chest X‐ray and CT of case 2 (A) Chest X‐ray on admission showed an increased cardiothoracic ratio. (B) CT on admission revealed thick subcutaneous fat surrounding the thorax and no infiltration in the pulmonary fields. (C) Chest X‐ray on day 11 showed extensively increased density in the bilateral lungs due to acute respiratory distress syndrome. (D) CT on day 11 revealed extensive infiltration in the pulmonary fields

A 45‐year‐old man with dyslipidemia who worked as an event hall staff was admitted to our hospital in the fall of 2021 with complaints of fatigue, lethargy, and leg edema for 2 weeks. As the pandemic reduced his working days, he increasingly stayed at home. He was 173 cm tall, weighed 180 kg, and had a BMI of 60.1 kg/m^2^. He had a history of hospitalization to reduce his weight twice, at ages 24 and 36. Arterial gas analysis on room air revealed AHRF (pH; 7.30, PaCO_2_; 80.3 mmHg, PaO_2_; 32.6 mmHg, ${\mathrm{HCO}}_{3}^{-}$; 38.4 mmol/L). On day 1, chest radiography and CT showed cardiomegaly and thick subcutaneous fat surrounding the thorax but no pulmonary infiltration (Figure 2A, B). Although echocardiographic evaluation was not sufficient due to obesity, brain natriuretic peptide (BNP) was elevated (350.5 pg/mL) and heart failure coexisted. Furosemide was administered and BiPAP was initiated (IPAP: 24 cmH_2_O, EPAP: 8 cmH_2_O, mode: spontaneous/timed, back‐up rate: 12/min, inspiratory time: 1 s). However, respiratory failure progressed rapidly, mechanical ventilation was needed, and antibiotics were started. The next day, he developed a fever of >38°C. A tracheotomy was performed on day 7. Chest radiography and CT showed acute respiratory distress syndrome (ARDS) on day 11 (Figure 2C, D). The patient died of respiratory failure on day 13.

### Case 3

**FIGURE 3:**
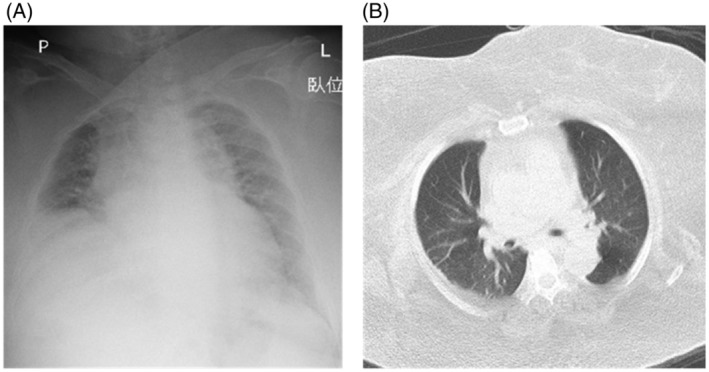
Chest X‐ray and CT of case 3 (A) Chest X‐ray on admission showed an increased cardiothoracic ratio. (B) CT on admission revealed thick subcutaneous fat surrounding the thorax and no obvious infiltration in the pulmonary fields

A 77‐year‐old woman with hypertension was brought to our emergency room. In February 2022, she became immobile due to lumbago after falling at home. A few days later, in the morning, her husband noticed that she had become lethargic and somnolent. She was 157 cm tall, weighed 123 kg, and had a BMI of 49.9 kg/m^2^. Arterial gas analysis on room air revealed AHRF (pH; 7.26, PaCO_2_; 72.2 mmHg, PaO_2_; 33.6 mmHg, ${\mathrm{HCO}}_{3}^{-}$; 30.9 mmol/L). Chest radiography and CT showed no pulmonary lesions, but thick subcutaneous fat surrounded the thorax (Figure 3A, B). BNP was elevated (248.8 pg/mL) and heart failure coexisted. Respiratory management with BiPAP (IPAP: 18 cmH_2_0, EPAP: 8 cmH_2_0, mode: spontaneous/timed, back‐up rate: 12/min, inspiratory time: 1 s) was initiated, but respiratory failure was prolonged. We gradually extend CPAP time during the day and tried to switch from BiPAP to CPAP, but within a few days, she had a re‐exacerbation and had to return to BiPAP several times. It took 2 months to fully switch from BiPAP to CPAP. After 6 months of hospitalization with persistent rehabilitation and calorie restriction, she finally succeeded in losing 16 kg of weight and respiratory failure was improved (pH; 7.44, PaCO_2_; 47.7 mmHg, PaO_2_; 64.5 mmHg, ${\mathrm{HCO}}_{3}^{-}$; 31.5 mmol/L [room air]). She was able to walk short distances and was discharged home on CPAP at night.

### Case 4

**FIGURE 4:**
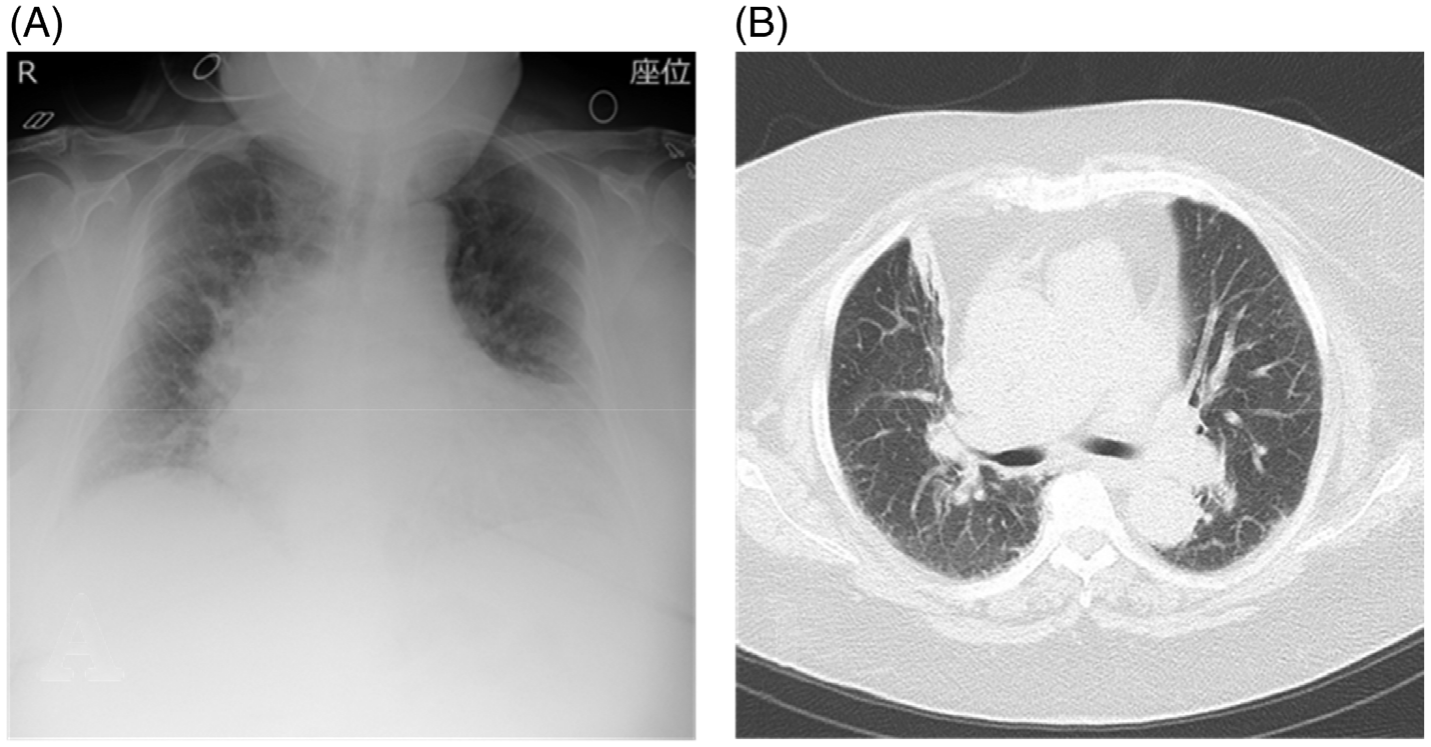
Chest X‐ray and CT of case 4 (A) Chest X‐ray on admission showed an increased cardiothoracic ratio. (B) CT on admission revealed thick subcutaneous fat surrounding the thorax and no obvious infiltration in the pulmonary fields

A 77‐year‐old woman with diabetes mellitus had difficulty walking because of obesity and knee osteoarthritis. In the spring of 2022, she fell at home and could not stand because of knee pain. A few days later, her family noticed her impaired consciousness and brought her to our hospital. She was 145 cm tall, weighed 99.3 kg, and had a BMI of 47.2 kg/m^2^. The patient developed AHRF (pH; 7.28, PaCO_2_; 71.1 mmHg, PaO_2_; 145.0 mmHg, ${\mathrm{HCO}}_{3}^{-}$; 34.2 mmol/L [on face mask O_2_ 6 L]). Chest radiography and CT scan showed no pulmonary lesions, but thick subcutaneous fat surrounded the thorax (Figure 4A, B). BiPAP (IPAP: 10 cmH_2_O, EPAP: 4 cmH_2_O, mode: spontaneous/timed, back‐up rate: 12/min, inspiratory time: 1 sec) was initiated, and her consciousness improved. BiPAP was continued all day for 11 days and then gradually switched to CPAP. She worked on calorie restriction and rehabilitation while using CPAP at night, and the respiratory failure gradually ameliorated (pH; 7.42, PaCO_2_; 46.7 mmHg, PaO_2_; 81.5 mmHg, ${\mathrm{HCO}}_{3}^{-}$; 29.9 mmol/L [room air]). After 2 months of hospitalization, she successfully lost 19 kg and was transferred to another hospital for continued rehabilitation and returned home 2 months later.

## DISCUSSION

AHRF in OHS has been relatively well reported in the Western world but is not well recognized in Japan.^5^, ^6^ The incidence of obesity in Japan has been steadily increasing, with a 30% increase in the rate of obesity with BMI ≥30 kg/m^2^ over the past decade (from 3.4% in 2010 to 4.5% in 2019).^7^ The prevalence of OHS increases with the degree of obesity increases.^2^ The COVID‐19 pandemic had a significant impact on social life. In our hospital, we experienced four cases of OHS‐associated AHRF in about a year; we had rarely experienced such cases before the COVID‐19 pandemic (Table 1). In case 1, scoliosis may be involved in sleep‐disordered breathing, which was treated by CPAP before admission. In the other three cases, no respiratory disease was diagnosed before admission. All patients exhibited changes in consciousness at the time of admission, but no complaint of dyspnea was reported. The absence of dyspnea may be associated with an impaired central respiratory drive.^8^ Heart failure or respiratory infection are known as the main causes of OHS‐associated AHRF.^5^, ^6^ On admission, all patients had no sign of infection, including fever, leukocytosis, or pulmonary infiltration. Echocardiography was performed in all patients, but thick fat made observation inadequate. However, heart failure was present in cases 2 and 3, although the causal relationship to the onset of AHRF is unclear. The two elderly women had a history of falling, which preceded the exacerbation. The length of hospital stay for all patients was prolonged, apart from the patient who died. In case 1, severe hypoxemia and ICU management resulted in lower extremity paralysis due to critical illness myopathy, which required prolonged hospitalization for rehabilitation. In case 2, heart failure coexistence may have been related to the rapid progression of respiratory failure. Intubation was inevitable, and the onset of ARDS caused by pneumonia was fatal. The two elderly women had prolonged respiratory failure and prolonged rehabilitation; thus, they required prolonged hospitalization before being able to live independently at home.

**TABLE 1:** Characteristics and outcomes of patients

Age	Sex	BMI (kg/m^2^)	Comorbidity	Trigger of precipitation	Complaints	Respiratory management	Outcome (observation period)
24	Male	45.8	Prader‐Willi syndrome diabetes scoliosis	constipation weight gain	appetite loss dizziness	BiPAP/CPAP	alive at facility (22 months)
45	Male	60.1	dyslipidemia	Unknown	fatigue lethargy	BiPAP/ventilator	died of ARDS (2 weeks)
77	Female	49.9	hypertension	Fall	impaired consciousness	BiPAP/CPAP	alive at home (14 months)
77	Female	47.2	Diabetes	Fall	impaired consciousness	BiPAP/CPAP	alive at home (10 months)

Abbreviations: ARDS, acute respiratory distress syndrome; BiPAP, bilevel positive airway pressure; BMI, body mass index; CPAP, continuous positive airway pressure.

Although many patients with OHS often live in a subclinical state without obvious clinical signs, the COVID‐19 pandemic could have had a fatal impact on the lifestyles of some patients, leading to the onset of AHRF. Many studies have shown decreases in physical activity due to the pandemic, raising concerns about the increased incidence of obesity and its impact on health.^9^ For the two female patients, decreased physical activity may be associated with muscle weakness and induced falls, which likely contributed to their AHFR as supine positioning has been shown to worsen respiratory function in patients with obesity due to ventilatory to perfusion ratio mismatch and overstretching of the diaphragm.^8^, ^10^ OSA patients with OHS have been demonstrated to have a higher rate of postoperative respiratory failure than OSA patients without OHS.^11^ Orthopaedic surgery and trauma were reported to induce AHRF in patients with OHS.^5^, ^12^ Some Japanese studies have shown that the pandemic reduced physical activity and increased frailty in the elderly.^13^, ^14^ Since women with OHS are known to be significantly older than men with OHS, older women with OHS may be more susceptible to the effects of the pandemic.^15^ Falls can be a significant risk factor in older patients. The onset of AHRF is sometimes fatal, greatly compromising the patient's quality of life and placing a significant burden on healthcare professionals.

In conclusion, this report described four patients with OHS who experienced AHFR during the COVID‐19 pandemic. Previously, we have rarely experienced cases of OHS‐associated AHRF in our region of Japan; and this may be due to pandemic‐related changes in lifestyle causing increased obesity, deconditioning, and falls.

## AUTHOR CONTRIBUTIONS

Sato Nakamura wrote the manuscript. Akihiro Kikushima, Atsushi Muto, Yoshifumi Suda, and Kazuki Gohara were involved in data analysis. Yukio Kawagishi and Shinichi Takeda reviewed and edited the manuscript.

## CONFLICT OF INTEREST STATEMENT

None declared.

## ETHICS STATEMENT

The authors declare that appropriate written informed consent was obtained for the publication of this manuscript and accompanying images.

## Data Availability

The data that support the findings of this study are available from the corresponding author upon reasonable request.
